# Animals can assign novel odours to a known category

**DOI:** 10.1038/s41598-017-09454-0

**Published:** 2017-08-21

**Authors:** Hannah F. Wright, Anna Wilkinson, Ruth S. Croxton, Deanna K. Graham, Rebecca C. Harding, Hayley L. Hodkinson, Benjamin Keep, Nina R. Cracknell, Helen E. Zulch

**Affiliations:** 1 School of Life Sciences, University of Lincoln, Joseph Banks Laboratories, Lincoln, LN6 7DL UK; 2 School of Chemistry, University of Lincoln, Joseph Banks Laboratories, Lincoln, LN6 7DL UK; 3 Defence Science and Technology Laboratory, Fort Halstead, Sevenoaks, Kent, TN14 7BP UK

## Abstract

The ability to identify a novel stimulus as a member of a known category allows an organism to respond appropriately towards it. Categorisation is thus a fundamental component of cognition and an essential tool for processing and responding to unknown stimuli. Therefore, one might expect to observe it throughout the animal kingdom and across sensory domains. There is much evidence of visual categorisation in non-human animals, but we currently know little about this process in other modalities. In this experiment, we investigated categorisation in the olfactory domain. Dogs were trained to discriminate between 40 odours; the presence or absence of accelerants formed the categorical rule. Those in the experimental group were rewarded for responding to substrates with accelerants (either burnt or un-burnt) and inhibit responses to the same substrates (either burnt or un-burnt) without accelerants (S+ counterbalanced). The pseudocategory control group was trained on the same stimuli without the categorical rule. The experimental group learned the discrimination and animals were able to generalise to novel stimuli from the same category. None of the control animals were able to learn the discrimination within the maximum number of trials. This study provides the first evidence that non-human animals can learn to categorise non-biologically relevant odour information.

## Introduction

Across the animal kingdom, organisms need to be able to evaluate their current situation and respond to events in terms of their likely consequences (e.g. fleeing from a predator or approaching a potential mate)^1^. If the specific event encountered is novel, then the animal must select information from its previous experience and use this as a basis for an appropriate response. Categorisation is the ability to treat comparable but non-identical stimuli as equivalent by responding to them according to the category to which they belong^2^. Thus, the ability to identify a novel stimulus as a member of a known category allows the organism to respond towards it in an appropriate way^2^.

Herrnstein and Loveland^3^ pioneered the study of categorisation in non-human animals (hereafter animals) and their seminal work paved the way for the vast amount of research which has furthered our understanding of categorisation in the visual domain across a wide range of species^4–7^. Animals can learn about stimuli in their environment in different ways. A category-specific rule requires the animal to extract and combine features common to most (or maybe even all) instances of a class of stimulus and then to react in the same way to all stimuli possessing those features^8^. Alternatively, animals can learn the individual features and outcome of every individual stimulus (rote learning). The classic test to distinguish between rote learning and use of category-specific features is to use a “pseudo-category” control e.g. refs 9–11. In this paradigm, two groups of animals are trained. The same stimulus set is used for both groups but one group of animals are rewarded on the basis of a perceptual rule (the experimental group), whereas for the other group, stimuli are assigned at random so that no perceptual rule can be used for classification (the pseudo-category group; also termed the control group). Evidence suggests that different pathways may be used to remember categorical and rote learned information^9^; this is reflected in memory retention abilities, with more efficient learning and superior retention being evident when categorical processing is involved.

Research investigating odour discrimination in animals generally examines the role of training stimulus properties on detection and generalisation. This includes investigating the impact of exposure to specific odour components on the ability to generalise to mixtures of the same components e.g. refs 12, 13, generalisation versus discrimination of related molecules by odour e.g. ref. 14 or detection of odours in specific environments e.g. ref. 15. The findings suggest that, in general, similarity between odours enhances generalisation and reduces the ability of the animal to discriminate^16–18^. Taken together, this suggests that stimuli that are similar in some aspect(s) are likely to promote category formation.

The only work, to date, that suggests the animals may be able to categorise on the basis of odour information comes from research investigating the cognitive mechanisms underlying individual odour recognition in hamsters^19, 20^. Using a habituation-discrimination procedure, male hamsters were habituated to specific scent or secretion from one female (female A) before being presented with a different scent from the same female and another female (female B). The results revealed that the males can discriminate between the two individuals, with animals exploring the odour of the non-habituated female (female B) more. Crucially, the effect did not occur if the males did not have direct experience of the individuals, suggesting that odour similarity did not control the behaviour, but that the animals had formed a concept (or category) of each individual^20^.

The ability of animals to learn to categorise non-biologically relevant odours, differing according to a perceptual rule (similar to those used to investigate categorisation in the visual domain^3, 5, 9^) has never been investigated in animals. This lack of knowledge is remarkable given the fundamental importance of this stimulus modality in the success of many species. It is therefore likely that animals will demonstrate an ability to categorise odours.

Accelerants are an ideal odour group for testing this ability as the group contains a wide range of stimuli with similarities in chemical profile^21^ and dogs are frequently taught to discriminate these substances in their training for forensic fire investigation.

In this study, dogs were pre-trained to differentially respond to four odours (two S+ and two S−) using a go/no-go paradigm, in which they needed to offer a behavioural response (e.g. sit) to the positive stimuli and inhibit that response to the negative odours. Upon reaching a performance criterion they started the experiment. For this, dogs were pseudorandomly assigned to either an experimental or control group. All animals received 40 training stimuli, which comprised of an array of substrates. A range of accelerants were added to half of the substrates. Animals in the experimental group were differentially reinforced for responding to the presence of accelerant, burnt or unburnt, compared to absence of accelerants (categorical rule, S+ counterbalanced across animals). The same substrate could potentially be both a positive or a negative stimulus, depending on the presence or absence of accelerants. Control group dogs were trained using a pseudocategory, they were presented with the same stimuli but without the categorical rule (S+ and S− had a variety of substrates with and without accelerants, specific training stimuli counterbalanced across dogs).

After reaching a learning criterion, animals received a generalisation test to examine whether their learned response could be transferred to novel stimuli. If animals are able to categorise odour information then we predict that those in the experimental group would both learn the discrimination faster, generalise better to novel odours and retain the information over a substantial period of time.

## Results

### Pre-training

Eleven dogs completed pre-training (see supplementary information for individual dog details). They were then assigned to the experimental (n = 6) or control group (n = 5) on the basis of pre-training performance. This was to ensure that there was no significant difference between experimental and control groups in speed of learning in the final phase of pre-training (trials to criterion: experimental group (n = 6) 223 ± 135.75; control group (n = 5) 196 ± 96.59; t(9) = 0.376, p = 0.715.

### Training

All animals in the experimental group progressed to the final phase of training on the experimental odours (433.33 ± 246.14 trials). Only one animal in the control group reached this stage; it took 530 trials.

In the final phase of training, in which the experimenter was blind to any odour information, four experimental group animals reached criterion (537.50 ± 289.41 trials). However, the control animals failed to reach learning criterion within 101 sessions (1010 trials).

**Figure 1:**
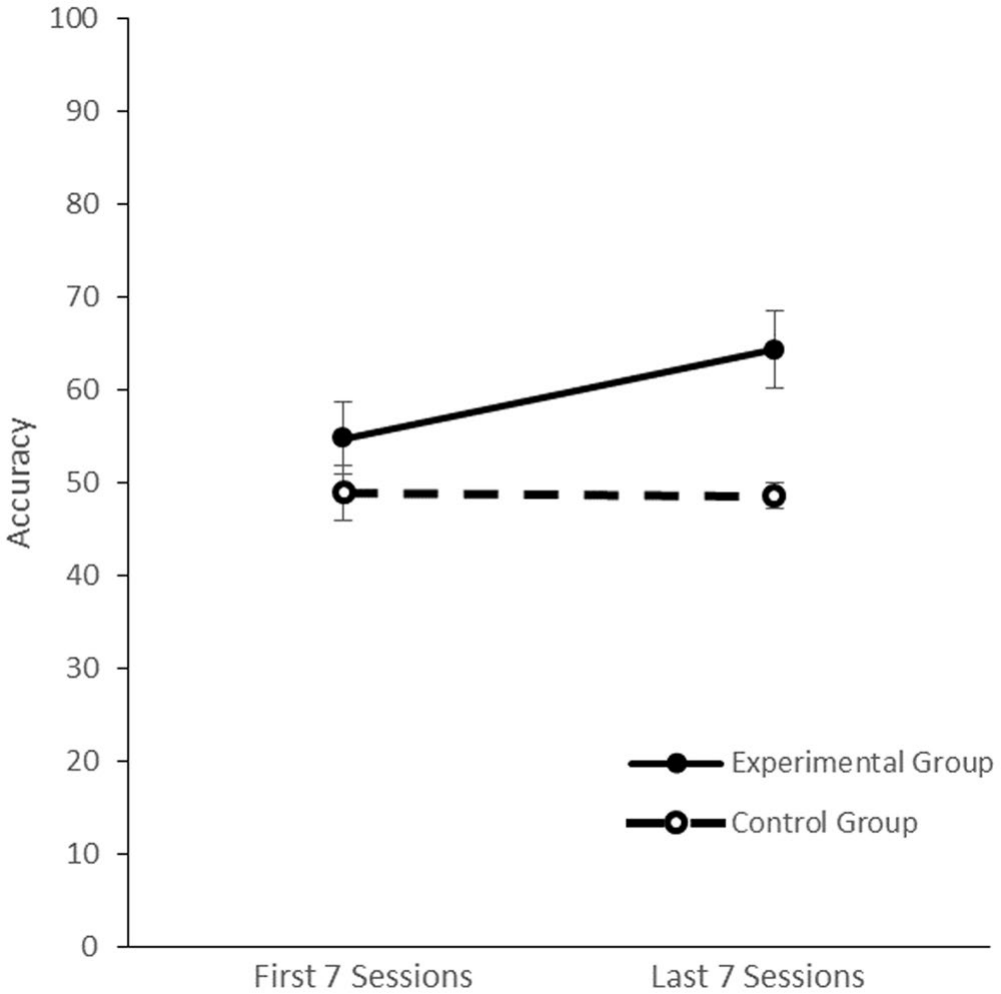
Training data for experimental (n = 6) and control (n = 5) group: % accuracy of responses to the odour stimuli in the first seven vs. last seven sessions.

As the fastest dog reached criteria in 14 sessions, we compared the data between the first seven and last seven sessions. A mixed model ANOVA revealed a significant interaction between group and time, with the experimental group showing a greater increase in % accuracy from first seven to last seven sessions (F (1,9) = 9.27, p = 0.014, ηp^2^ = 0.51; Fig. 1).

### Generalisation test

The four experimental group dogs that learned the task received a generalisation test in which they were presented with novel stimuli which were designated as belonging to either an accelerant or non-accelerant category. No control animals reached this stage. One of the experimental dogs was withdrawn during testing as she failed to engage on 7 of her last 10 generalisation sessions.

**Figure 2:**
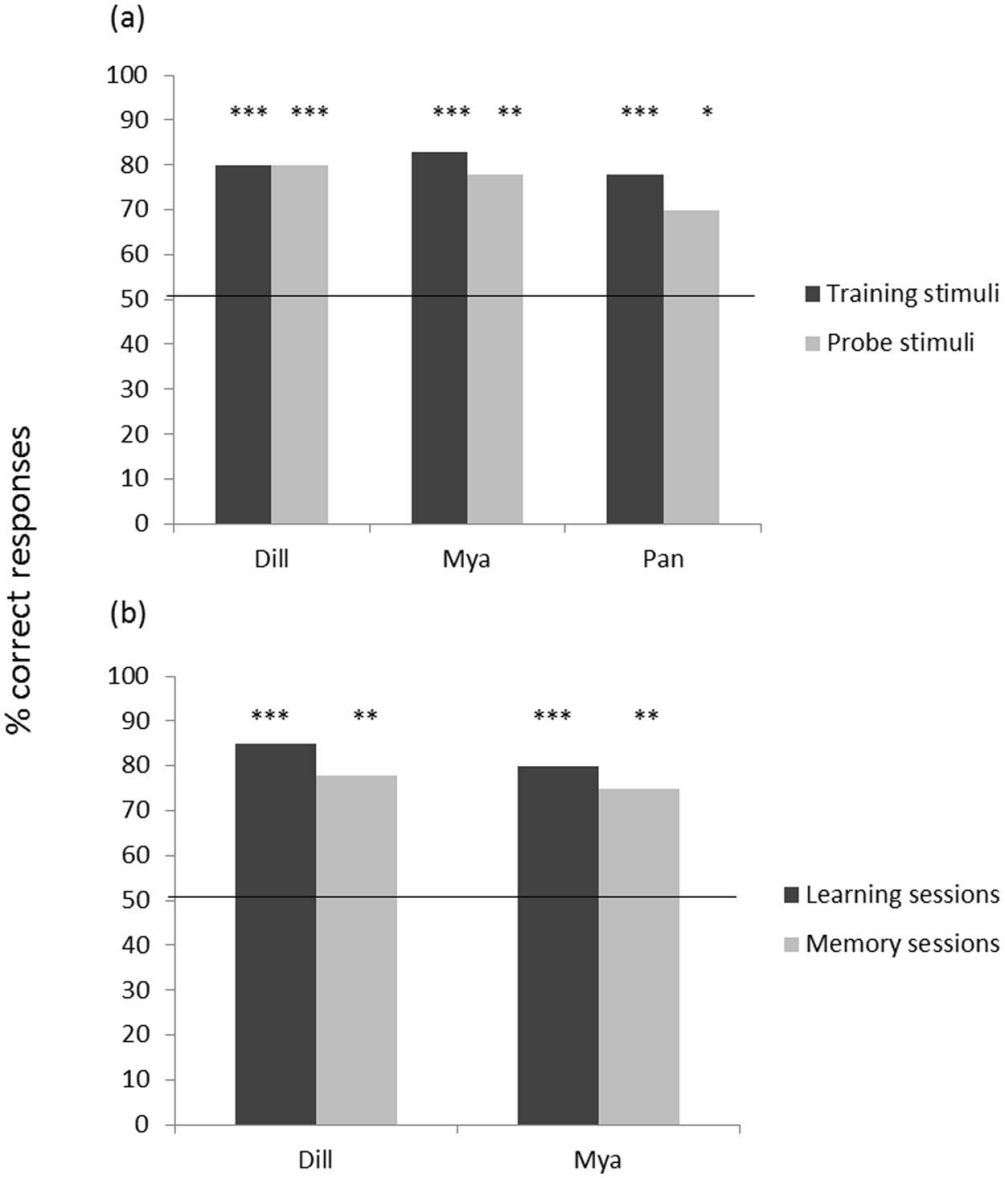
Percentage of correct responses in generalisation test (**a**) and memory test (**b**). Learning sessions represent the performances on the final training session prior to the tests taking place. The solid line represents chance level (50%). Binomial test: *P < 0.05; **P < 0.01; ***P < 0.001.

All three animals that completed testing successfully transferred the learned discrimination to novel stimuli within the trained category as assessed by means of a two-tailed binomial test comparing performance to chance (Dill p < 0.001, Mya p < 0.01, Pan p < 0.05, Fig. 2a).

### Memory test

To investigate information retention, animals were re-tested in a novel location more than 6 weeks after the last exposure to the training stimuli. Two dogs completed the memory test and performance was significantly above chance as assessed by means of a two-tailed binomial test (p < 0.01 for both animals, Fig. 2b).

## Discussion

This study represents the first demonstration that animals are able to categorise odours outside the sphere of individual recognition. Processing novel stimuli both efficiently and effectively is essential for survival^1^. The ability to categorise is considered to be fundamental to this^2^. We showed that the categorisation group were able to successfully meet learning criteria, whereas all control dogs failed. Further, the experimental group animals were able to generalise to novel exemplars of the category and were able to retain this information following six weeks of non-exposure.

As the control animals were not able to progress beyond the early stage of training, it is not possible to say whether they were incapable of learning the stimuli, or whether they simply did not have enough exposure. It is also not possible to say whether they would be able to generalise should they have reached learning criterion. However, it is clear that despite no differences in general learning ability between the groups as evidenced by pre-training performance, the task was considerably more difficult without a categorical rule. This has implications in the field of working dog training as it implies that current training methods which involve sequential rote learning may not be best practice.

Given the complexity of the odour stimuli used in this study, it remains unclear exactly what features of the odour profile the dogs were extracting in the probe stimuli and how they were able to map these against previous examples (i.e. trained stimuli) from the same category. However, it is clear that the ability of the experimental animals to learn the categorical rule was not a result of the feature positive effect^22^ as the category rule was counterbalanced across animals and, of those that achieved learning criteria, two animals had the accelerant as S+ and two had the absence of accelerant as S+.

In summary, we demonstrate for the first time that animals have the ability to learn to categorise non-biologically relevant odours when these are presented as a learning set. Further, they were able to generalise this behaviour to novel stimuli that contained similar perceptual properties to the training stimuli. These findings add substantially to our understanding of how animals process olfactory information and suggest that categorisation is fundamental to processing stimuli across modalities.

## Methods

This research was approved by the ethics committee of the School of Life Sciences, University of Lincoln (HZ2013-001). The work complied with UK requirements for research on animals.

Eleven mesocephalic pet dogs passed the pre-training (Table 1 supplementary information). Informed consent was obtained from their owners.

### Pre-training

Four essential oils were used for pre-training. Each animal was allocated two positive (S+) and two negative (S−) stimuli (counterbalanced across subjects) and trained to make a response (e.g. sit) to S+ and inhibit responding (e.g. remain standing) to S−. The stimuli, which varied in strength, were presented in a metal canister placed close to the animal’s nose.

Stimuli were presented in sessions of 10 trials (half S+ and half S−). The order of presentation was pseudo-random to ensure that no more than 3 S+ or S− were presented consecutively. Initially responses were verbally cued by the researcher, this was faded during training. Responses were scored as either: True Positive (TP: the dog indicates the presence of the S+ and the S+ is present), True Negative (TN: the dog indicates the absence of the S+ and the S+ is not present, in this case the S− would be present), False Positive (FP: the dog indicates the presence of the S+ when the S+ is absent, in this case the S− would be present) or False Negative (FN: the dog fails to indicate the presence of the S+ when the S+ is present).

### Pretraining Procedure

Two experimenters were present at every session. During a session, experimenter one presented the odour stimuli to the animal and reinforced correct responses with a food reward, or, punished incorrect responses by withholding the food, moving away from the immediate testing for 2–10 seconds and not engaging with the dog during this time. Experimenter two (who was behind a solid barrier and could not be seen by the animal) handed the stimuli to experimenter one and recorded responses. Dogs were pretrained with a default position (e.g. stand) and a response behaviour (e.g. sit) until the default position was maintained up to 10 seconds in the absence of any given cue and the response behaviour was under stimulus control. For the first 30 trials, presentation of S+ was immediately followed by a verbal cue for the subject’s response behaviour (e.g. sit). Correct responses to S+ were marked using a conditioned reinforcer then followed by a primary (food) reinforcer. Presentation of S− stimuli were immediately followed by the conditioned reinforcer (verbal: ‘good’) then primary (food) reinforcer for a non-response (e.g. remaining in stand). FP and FN responses were marked with the conditioned negative punisher, followed by the experimenter moving away from the immediate training area for approximately 3 seconds. After the first 30 trials, verbal cues for the response to S+ were reduced until animals were responding to the presentation of the stimulus alone. Once animals met criterion of ≥80%TP S+ and ≥80%TN without verbal cues within one session, all further sessions were conducted blind to the odour presenter.

During blinded sessions, the second experimenter observed the dog via live video link and verbally marked correct/incorrect responses using the conditioned reinforcer/conditioned negative punisher, the first experimenter then reinforced/punished the dog as appropriate. This was the final phase of pre-training.

Learning criteria to move to the main experiment were defined as follows: Minimum 10 blind sessions on pre-training odours; 3 out of 4 consecutive sessions ≥60%TP and ≥60%TN, with the 4th at least ≥40%TP and ≥40%TN.

### Experimental Stimuli

A total of 60 stimuli were created, they consisted of 15 substrates, with or without accelerant, burnt or unburnt. Fifteen different accelerants were used, these included representatives of the different classes, light, medium and heavy petroleum. Samples were prepared every 7 days. Substrates varied in size (approx. 2 cm^2^–4 cm^2^), and, where present, variable amounts of accelerant (0.5ml–2ml) was pipetted onto substrates. Half of the samples were burnt until the flame extinguished naturally. Once prepared, samples were placed in the presentation pots. Each animal received 40 stimuli as training odours; 20 S+ and 20 S− and 10 stimuli as test odours 5 S+ and 5 S−. The remaining stimuli allowed for a wider pool from which to select the sample sets.

Animals were pseudorandomly allocated to a condition, these were matched for learning speed during pre-training. Animals in the experimental group were differentially reinforced for responding to the presence of accelerant, which could be burnt or unburnt, compared to absence of accelerants (categorical rule, S+ counterbalanced across animals). Control group dogs were trained with the same stimuli but as a pseudocategory (without the categorical rule. S+ and S− had a variety of substrates with and without accelerants, the specific stimuli used were counterbalanced across dogs).

### Experimental Procedure

Each animal was trained and tested on a range of days of the week in order to introduce variety in the age of odour. The stimulus set was matched between groups and the order of presentation was counterbalanced across animals and pseudo-randomised to ensure that each stimulus occurred once in every 4 sessions.

The experimental procedure was the same as described for pre-training except that, after exposure to all training stimuli, dogs were given a blind session in which the odour presenter was unaware of contingencies associated with the stimuli. Feedback was given by a second experimenter who was behind a screen and watched the dog’s response via a computer monitor. This was to ensure that the dogs could not use any spurious cues from the presenter when making their decision. After this, every fourth session was blind. When ≥80% TP and ≥80% TN was reached on a single blind session, dogs received only blind sessions. Upon reaching learning criteria; this required exposure to every stimulus at least twice in blind sessions and performance of ≥80% TP and ≥80% TN on 3 out of 5 consecutive blind sessions, animals were given the generalisation test.

### Generalisation tests

In each test session, two probe stimuli (to which the animal had no prior exposure) were pseudo-randomly intermixed with the 10 training stimuli. They were pseudorandomly inserted in trials 2–9, separated by at least one training stimulus balanced for S+/S− presented first and never presented in a row of three of more S+/S− overall. Dogs received no differential feedback on probe stimuli. Animals received 4 repetitions of each of the 10 probe stimuli. If an animal did not perform at minimum criteria on the training trials in a test session then the sessions were re-run.

### Memory tests

Animals were re-tested in a new location at least 6 weeks after the last exposure to the training stimuli. After limited exposure to pre-training odours, animals were tested on their training stimuli for 4 sessions. This equated to one presentation of each of the 40 training odours. No stimuli were repeated and thus stimuli were differentially reinforced.

### Data Analysis

Data were checked for normality using Kolgomorov–Smirnov tests. Response performance within learning sessions was reported as % accuracy: ((TP-FP) + 1)/2*100 and a mixed model ANOVA was used to compare speed of learning between experimental and control groups over time. The discrimination performance in generalisation and memory tests was assessed by means of two-tailed binomial tests.

### Ethical Statement

This research was approved by the ethics committee of the School of Life Sciences, University of Lincoln. Applicable national guidelines for the care and use of animals were followed.

## Electronic supplementary material

Supplementary Information
