# Supplementary material for: Animals can assign novel odours to a known category
Source: Sci Rep. 2017 Aug 21;7:9019. doi: 10.1038/s41598-017-09454-0 (PMC5567083; doi:10.1038/s41598-017-09454-0)
Supplement: Supplementary file 1 — Supplementary Information [file 41598_2017_9454_MOESM1_ESM.doc]

**Animals can assign novel odours to a known category**

Hannah F. Wright^1^, Anna Wilkinson^1*^, Ruth S. Croxton^2^, Deanna, K. Graham^1^, Rebecca, C. Harding^1^, Hayley L. Hodkinson^1^, Benjamin Keep^1^, Nina R. Cracknell^3^, Helen E. Zulch^1^

^1^School of Life Sciences, University of Lincoln, Joseph Banks Laboratories, Lincoln, LN6 7DL, UK

^2^School of Chemistry, University of Lincoln, Joseph Banks Laboratories, Lincoln, LN6 7DL, UK

^3^Defence Science and Technology Laboratory, Fort Halstead, Sevenoaks, Kent, TN14 7BP, UK

^*^Author for correspondence (awilkinson@lincoln.ac.uk)

Table S1

| **Group** | **Animal** | **Age (years)** | **Sex/neuter** | **Breed** |
| --- | --- | --- | --- | --- |
| Experimental | uMoya | 1 | F | Labrador |
| Experimental | Dill | 1 | M | Large Munsterlander |
| Experimental | Kess | 2 | F | Labrador |
| Experimental | Pan | 4 | F | Siberian Husky |
| Experimental | Mya | 4 | F | Working Cocker Spaniel |
| Experimental | Mia | 6 | F | Labrador |
| **Average** |  | **3** |  |  |
| Control | Jupiter | 2 | M | Labrador |
| Control | Lily | 2 | F | Pointer cross |
| Control | Toffee | 4 | F | Spaniel cross |
| Control | Spook | 6 | F | Border Collie |
| Control | Meg | 6 | F | Labrador |
| **Average** |  | **4** |  |  |

Table caption:

Table S1: Subjects completing procedural training: Group; age; sex (M=male, F=Female); breed.
